# Obstetrics

**Published:** 1879-11

**Authors:** 


### OBSTETRICS.

ALTERATIONS OF THE DEAD-BORN FŒTUS. M. Dénarié. (*France Médicale*, Sept. 13, 1879.)

The fœtus dead in the mother's womb is not always immediately expelled; it may remain a greater or less time in the uterine cavity, and there undergoes various modifications, described under the term maceration. Maceration is not putrefaction: the fœtus is in a closed cavity, where it has no contact with the air, which is the important element in putrefaction. Maceration is revealed by several phenomena observed in the fœtus and its annexes: placenta, cord, amniotic fluid, membranes.

1. *Fœtus.* The principal characters presented in a macerated fœtus, which permit it to be recognized from a fœtus dead after birth, are: Absence of cadaveric rigidity, softness of ligaments, epidermic desquamation, change of color in the skin and organs, and softening of the latter.

The first phenomenon which strikes us in examining a dead born fœtus is the complete *absence of cadaveric rigidity.* To be of importance, however, this phenomenon must be observed before the fœtus has undergone malaxations, or before putrefaction has set in, for rigidity disappears soon under the influence of both these causes.

Besides this absence of rigidity, which comes from muscular relaxation, great softness of the ligaments is seen, hence extreme mobility of all the articulations, and in particular of the sutures of the bones of the skull. These last, extremely mobile, give on palpation a special sensation compared to that of a bag of nuts. About these the hairy scalp becomes too large, presents folds at various points. This phenomenon may serve to diagnosticate the death of the fœtus while it is still in the uterine cavity, by means of the touch.

The epidermic desquamation is an important sign. It may be produced either spontaneously or accidentally. Spontaneous desquamation is due to the formation of phlyctenæ, produced by an exudation of serum; these break and leave the derma exposed. Accidental desquamation is that which results from friction during delivery, or from handling afterward. The degree of desquamation varies according to the length of time since the death of the fœtus. When the death is recent, it is necessary to pinch the skin strongly to produce it; when long dead, on the contrary, it is produced by the slightest contact. From this fact we may determine the probable date of death. For this the progress of desquamation should be studied upon a fœtus recently dead, macerated in a liquid for some time, making daily observations of the epidermis.

In the desquamated parts the derma, exposed to the air, takes, at the end of some hours, a bright red color, due probably to the action of the oxygen of the air upon the hematine; at the same time the surface hardens from drying.

*The coloration of the fœtus* furnishes very valuable signs. At the beginning a yellow coloration is observed, due to the meconium, which always escapes at the moment of death and mixes with the amniotic fluid. But after some time this coloration disappears, to give place to a rosy or uniform slate-gray coloration, due to the blood which becomes altered, loses its properties and escapes out of the blood-vessels into the tissues. The slate-gray color probably results from a transformation of the hematine of the blood. It begins in the abdominal walls, where it presents itself in spots, which give to the abdomen an appearance resembling the belly of a frog. This similitude is increased by the flat form and flaccidity due the formation of post-mortem ascites, which is produced by the transudation of blood.

Beneath the skin, the plasma spreads into the meshes of the connective tissue, and forms either a rose-colored jelly or a white lardaceous substance.

The blood is more or less completely decomposed ; the globules become raspberry-like, are destroyed, and their coloring matter mixes with the plasma, which gives it a color varying from rose to brown-red, and which, changing with time, may even approach that of leather. In the large veins, as the vena cava, large clots are found.

All the organs finish by taking a uniform tint, the result of progressive imbibition of the altered sanguinolent liquid in which they are continually bathed. This coloration, however, does not appear everywhere in the same manner; thus the liver preserves for a greater or less time its yellowish color due to the bile, and the brain its white color.

At the same time that they become colored the organs soften. The softening commences with the brain, which then appears under the form of a rose-colored pulp. The lungs and muscles preserve their solidity longest; they sink in water; but as they are susceptible of being inflated we should not conclude positively if they float that the fœtus has breathed, for the insufflation may have been produced artificially.

The eyes become soft; sometimes the white of the eye can no longer be distinguished, it having taken on a red coloration ; the cornea is lusterless and turbid. At the end of some days there is found in the ocular cavity a sanguinolent liquid which has replaced the aqueous and vitreous humor. In cases long dead the eye-ball is competely flattened.

Microsopic examination of macerated fœtuses has been made by Virchow and by Lempereur. (Thèse, Paris, 1865.) The common character of alteration of all the organs is fatty degeneration.

In the muscles, striation is recognized, between which are seen fine fatty granulations; the cells of the liver are destroyed; their place being taken by a fine granular detritus. The blood at the beginning may present raspberry-like globules, but soon only amorphous elements and fatty cells are found.

It is generally impossible to diagnosticate the cause of death from examination of the fœtus; it is sometimes found in the placenta; but often it is found neither in the fœtus nor in its annexes, for it comes from the mother or from detachment of the placenta.

The epoch of death is very difficult to determine, for we have no data. Still we may establish several periods:

In the first period, which lasts about two days, the fœtus is colored yellow by the meconium. In the second period the yellow color disappears to give place to coloration by hematine. According as this coloration is more or less uniform and more or less deep, the death is more or less ancient. In the third period the sanguine color has completely disappeared, the fœtus is entirely of a grayish white; this state comes on at the end of a month.

2. *Placenta.* It presents a uniform color resembling that of the fœtus, and displacing the normal color. Nevertheless, in the parts where the mother's blood circulates, there are still found traces of maternal circulation; the blood has not yet lost its color, and recent unaltered clots are frequently encountered, though no traces of them are found in the fœtus itself. The normal consistence disappears to give place to great friability and flaccidity. With the microscope the villosities are found much larger than in the normal state, and are infiltrated with fatty granules. This augmentation of volume of the villosities due to infiltration leads to an increase of the total volume of the placenta.

3. *The Cord.* The cord, like the placenta is more flaccid and presents a uniform color like the fœtus, is smooth and infiltrated, flattened, and there is a disappearance of its spiral form.

4. *Amniotic Fluid.* During the first period it is colored by the meconium. At the second stage it takes on a reddish color and finally brownish, due to transudation of the blood of the fœtus through the tissues.

5. *The membranes* present the same change of color. In the first period they are strongly tinted yellow by the bile. In the second they become reddish. The amniotic fluid is the vehicle of the coloring matter. The membranes become more friable than in the normal state.

From these considerations result the following practical points:

1. When the amniotic fluid escaping from the bag of waters is colored by meconium or blood, death of the fœtus may be feared.

2. Epidermic desquamation of the fœtus is an incontestible proof of death *in utero.*

3. Débris of cord or placenta suffice to denote death before labor.

DOUBLE UTERUS WITH DOUBLE CONCEPTION. (*Gazette Obstétricale*, Sept. 5, 1879.)

Dr. Sotschawa, of Moscow, reports, in the St. Petersburg *Med. Woch.*, Jan., 1879, the case of a woman, aged 26 years, who called him on account of a hæmorrhage occurring during a third pregnancy. On examination he found two distinct vaginas, each one terminating in a uterus. The finger passed readily through the first of these, and he found an ovum presenting; the uterus seemed to correspond to about the second month of conception. The vagina of the other side (right) was narrower, but the neck could be reached, and appeared to belong to a uterus of three months. The hæmorrhage had its source in both uteri, and in consequence was considerable; an embryo of one month was extracted with the finger from the left uterus, and three days later a fœtus of three months was extracted from right uterus. The author observes that this case is not only remarkable for its rarity (only thirty cases being on record) but also because it is a proof of the possibility of superfœtation.

